# Age at diagnosis, diabetes duration and the risk of cardiovascular disease in patients with diabetes mellitus: a cross-sectional study

**DOI:** 10.3389/fendo.2023.1131395

**Published:** 2023-05-08

**Authors:** Xuelin Yao, Jie Zhang, Xiaoqian Zhang, Tian Jiang, Yi Zhang, Fang Dai, Honglin Hu, Qiu Zhang

**Affiliations:** Department of Endocrinology, First Affiliated Hospital of Anhui Medical University, Hefei, China

**Keywords:** diabetes mellitus, cardiovascular disease, diabetes duration, age at diagnosis, macrovascular complications

## Abstract

**Background:**

The purpose of the study was to evaluate characteristics and risk of cardiovascular disease (CVD) according to age at diagnosis and disease duration among adults with diabetes mellitus (DM).

**Methods:**

The association between age at diagnosis, diabetes duration and CVD were examined in 1,765 patients with DM. High risk of estimated ten-year atherosclerotic cardiovascular disease (ASCVD) was performed by the Prediction for ASCVD Risk in China (China-PAR) project. Data were compared with analysis of variance and χ2 test, respectively. Multiple logistic regression was used to determine the risk factors of CVD.

**Results:**

The mean age at diagnosis (± standard deviation) was 52.91 ± 10.25 years and diabetes duration was 8.06 ± 5.66 years. Subjects were divided into early-onset DM group (≤43 years), late-onset DM group (44 to 59 years), elderly-onset DM group (≥60 years) according to age at diagnosis. Diabetes duration was classified by 5 years. Both early-onset and longest diabetes duration (>15 years) had prominent hyperglycaemia. Diabetes duration was associated with the risk of ischemic stroke (odds ratio (OR), 1.091) and coronary artery disease (OR, 1.080). Early-onset group (OR, 2.323), and late-onset group (OR, 5.199), and hypertension (OR, 2.729) were associated with the risk of ischemic stroke. Late-onset group (OR, 5.001), disease duration (OR, 1.080), and hypertension (OR, 2.015) and hyperlipidemia (OR, 1.527) might increase the risk of coronary artery disease. Aged over 65 (OR, 10.192), central obesity (OR, 1.992), hypertension (OR, 18.816), cardiovascular drugs (OR, 5.184), antihypertensive drugs (OR, 2.780), and participants with disease duration >15 years (OR, 1.976) were associated with the high risk of estimated ten-year ASCVD in participants with DM.

**Conclusion:**

Age at diagnosis, diabetes duration, hypertension and hyperlipidemia were independent risks of CVD. Longest (>15 years) diabetes duration increased the high risk of ten-year ASCVD prediction among Chinese patients with DM. It’s urgent to emphasize the importance of age at diagnosis and diabetes duration to improve primary complication of diabetes.

## 1 Introduction

Diabetes mellitus (DM), commonly referred to as diabetes, is a chronic metabolic disorder caused by insufficient insulin production and/or insulin resistance resulting from both environmental and genetic components (1). Its prevalence increased dramatically over the last three decades. An estimated 536.6 million people in 20–79 year olds have DM in 2021, and this number is expected to reach 12.2% (783.2 million) by the year 2045 (1). Considering the health and economic consequences associated with DM (complications, obesity, mortality), there is huge interest in strategies to reduce DM prevalence (2, 3). Cardiovascular disease (CVD), including coronary artery disease, cerebrovascular disease, or peripheral arterial disease, is a macrovascular complication that mostly develops in patients with diabetes (4). The prevalence rate of CVD is higher in adults with diabetes than in those without diabetes (4). CVD mostly developed in the following cases: genetic predisposition, hypoglycaemia, during treatment, increased insulin resistance (4). Furthermore, CVD greatly contributes to morbidity and mortality among patients with DM. In addition, compared with patients without CVD, those diagnosed with the disease are particularly prominent among younger patients with hyperglycaemia and serious renal complications (5). Similar to the increase in DM prevalence, cerebrovascular disease prevalence has also increased globally and increasingly been recognised as cerebral macrovascular complications of type 2 diabetes mellitus (T2DM) (6). T2DM is associated with a 2.5-times increased risk of ischaemic stroke (7). Ischaemic stroke is also common among adults with prediabetes than among people with normoglycaemia, suggesting that cerebral infarcts processes start before the onset of diabetes (8, 9). In view of ageing populations and the growing prevalence of DM, DM prevention strategies are of paramount importance and identifying the mechanisms linking DM with CVD is key to reduce the macrovascular complications.

Previous data suggested that age, age at diagnosis and disease duration had varying effects on the risk of vascular complications in patients with DM (10–12). For example, Zoungas et al. reported that age or age-onset, and duration of diabetes were independently associated with macrovascular events and death, whereas only duration is independently associated with microvascular events (12). Subsequent studies confirmed age and duration of diabetes were strong risk factors for myocardial infarction, stroke, and heart failure (10). Furthermore, studies of people with T2DM using a propensity score-matched cohort analysis provide excellent insight into the early clinical course of diabetes, indicating the earlier onset of T2DM induce the higher risk of microvascular (11). Few studies evaluated clinical characteristics and macrovascular complications according to age at diagnosis and prolonged duration of diabetes among Chinese patients with DM (13). Therefore, the aim of our study is to investigate the relationship between onset age of diabetes and complications, and provide a better understanding of age at diagnosis on the risk of complications, and improved explanation of the modifiable risk factors to reduce the burden of macrovascular complications and T2DM.

## 2 Methods

### 2.1 Setting and study subjects

The current data analysis was based on the China National Diabetic Chronic Complications Study (China DiaChronic Study), which has been reported in detail previously (14). Briefly, a total of 53,401 Chinese adults with diagnosed diabetes ages 18–74 were recruited and completed baseline questionnaires and medical examinations between March 2018 and January 2020. The multistage sampling scheme (stratification, clustering, and random) was used to sample study participants based on the disease surveillance points (DSPs) started in 2013 from the China Chronic Disease and Risk Factors Surveillance (CCDRFS). The sampling details have been described elsewhere (15, 16). Stage 1, four DSPs were selected from 25 provinces, autonomous regions and municipalities, five DSPs were selected from Sichuan, Henan, Shandong, 2 to 3 DSPs were selected from Qinghai, Tibet, Xinjiang. In total, there are 122 study sites (65 urban and 57 rural DSPs) from the 2013 CCDRFS were invited to participate. Stage 2, four neighborhoods/villages were selected from each DSP, and a total of 260 neighborhoods and 228 villages were acquired. Stage 3, 58,560 Chinese adults with diagnosed diabetes ages 18-74 were recruited in the study after selecting 120 participants at each neighborhood/village by gender and age ratio.

**Figure 1:**
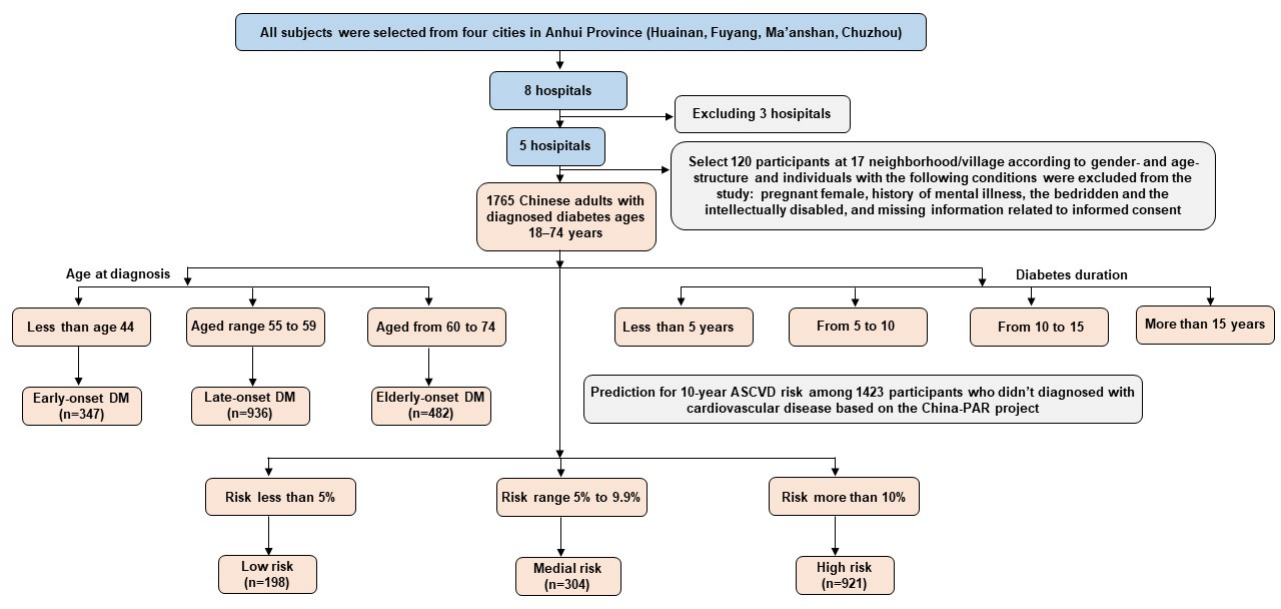
Flowchart of the subjects selecting in the study.

In this study, a total of 1,765 participants were recruited from four cities (Huainan, Fuyang, Ma’anshan, Chuzhou) in Anhui province. Each participant received a series of medical examinations including a general physical examination and biochemical tests of blood and urine sample, as well as a standard self-administered questionnaire survey. Moreover, to avoid the influence of confounding factors, we restricted our analysis to those participants aged 18−74 years, and who had resided in the study sites for at least 6 months during the 12 months prior to the survey. We also excluded those who reported the following conditions: pregnant female, history of mental illness, the bedridden and the intellectually disabled, and missing information related to informed consent. Information on personal age at diabetes mellitus diagnosis and diabetes duration was obtained by a self-administrated questionnaire. Diabetes duration was calculated by subtracting age at diagnosis from age at study baseline. Duration of diabetes was classified into four ordinal groups (≤5, >5−10, >10−15 and >15 years). For age at diagnosis, they were asked “When did you first diagnose diabetes?” Age at diagnosis was classified into three groups: less than age 44 (early-onset DM), aged range of 44 to 59 (late-onset DM), aged from 60 and 74 (elderly-onset DM). Furthermore, the China-PAR project was used to predict 10-year atherosclerotic cardiovascular disease (ASCVD) risk among Chinese population, the details have been described elsewhere (17–19). The Chinese prediction models directly included age, SBP, total cholesterol, HDL-C, current smoking, and diabetes. Four additional covariates (waist circumference (WC), geographic region, urbanization, and family history of ASCVD) met the pre-defined inclusion criteria based on relative integrated discrimination improvement (IDI) of 6% or greater. This tool is applicable to people aged 20 and above without history of cardiovascular and cerebrovascular diseases. Therefore, 1423 participants were selected in our study. According to predicted ASCVD risk, participants were divided into three categories: low risk (<5%), median risk (5%−9.9%), high risk (≥10%). Details of the workflow chart are presented in **Figure 1**. Personal identification was removed and remained anonymous when the data were released for this research. All participants provided written informed consent, and Ethical Review Committee (Approval No: 2018-010) approved the study, which was registered in the Chinese Clinical Trial registry (ChiCTR1800014432).

### 2.2 Estimation of sample size

The sample size calculation was estimated by using following formula.


$$n=\frac{\left(1-p\right)Z_{1-\alpha /2}}{\varepsilon^{2}p}$$

Z_1-α/2_ = 1.96, the prevalence of diabetes mellitus was 11.2% (p), the desired level of relative precision was 0.15 (ε) (20). Then, considering multicenter design and drop-out rate of 10% total sample size, hence the minimum sample required for conducting this study was found to be 1540.

### 2.3 Assessment of covariates

Information on sociodemographic characteristics collected in the baseline was used in the current data analysis, including age, sex, medical insurrance, marital status (single, married, lived together, divorced, separated and widowed), total annual family income and education level (primary school or below, middle school, and college, and university or above). Lifestyle factors including alcohol drinking (never, former, and current), cigarette smoking (never, former, and current), and family medical histories (yes, no) was collected at baseline by trained interviewers using semi-structured questionnaires. The general health examinations were performed by qualified personnel. Hyperglycaemia was defined as a glycosylated hemoglobin (HbA1c) >7.0% and fasting plasma glucose (FPG) >7.0 mmol/L (1). Body mass index (BMI) was calculated as weight in kilograms divided by height in meters squared. Obesity was defined as a BMI of ≥28 kg/m^2^ (21). WC was calculated by horizontal girth of waist through belly button. Central obesity was defined as WC ≥90 cm in men and WC ≥85 cm in women for Chinese (22). Resting blood pressure including systolic blood pressure (SBP) and diastolic blood pressure (DBP) was measured and fasting blood was drawn for laboratory assays of blood lipids, fasting glucose, glycosylated hemoglobin, hepatic function, renal function. Hypertension was defined as self-reported physiciandiagnosed hypertension or current use of antihypertensive medications or SBP ≥140mmHg/DBP ≥90 mmHg by the 2017 American College of Cardiology/American Heart Association guidelines (23). Myocardial infarction was defined as a history of physician-diagnosed myocardial infarction. Hyperlipidemia was defined as a history of physician-diagnosed hyperlipidemia or current usage of lipid-lowering medications or total cholesterol (TC) ≥5.20 mmol/L or triglycerides (TG) ≥1.70 mmol/L or high-density lipoprotein cholesterol (HDL-C)<1.0 mmol/L or low-density lipoprotein cholesterol (LDL-C) ≥3.4 mmol/L by the 2019 China Cholesterol Education Program (CCEP) expert advice for the management of dyslipidaemias to reduce cardiovascular risk (24). Information on medications collected in the baseline was used in our study, including antihypertensive drugs, cardiovascular drugs, lipid lowering medication, glucose lowering medication.

### 2.4 Ascertainment of the outcome

The presence of diabetic macrovascular complications (coronary artery disease and cerebrovascular disease) was assessed from the questionnaire of each participants. To collect the information, the participants were asked: “Have you ever been diagnosed as cardiovascular disease or ischemic stroke by physicians in medical institutions at county/district level or above?” Those participants who answered “yes” were then defined as having a event of cardiovascular disease or ischemic stroke. Furthermore, these patients were asked two questions: “When did you first diagnose these diseases?” “Did you hospitalized after you suffer from above mentioned disease?” A total of 234 ischemic stroke cases and 145 coronary artery disease cases were observed in this study.

### 2.5 Statistical analysis

Data were presented as mean (standard deviation) for continuous variables and percentages for categorical variables, respectively. χ2 test was employed to compare categorical variables between groups. Statistical differences between groups for continuous variables were compared with Mann-Whitney U tests or analysis of variance. Binary logistic regression analysis with forward selection was performed to identify independent factors associated with the risk of ischemic stroke and coronary artery disease. The results were presented as odds ratio (OR) and 95 percent confidence interval (95% CI), taking age at diagnosis of 60 to 74 years as the reference groups, based on the previous studies suggesting that the onset age after 60 years was less likely to have macrovascular and microvascular complications (11). Potential covariates included in the adjusted model were age, BMI, WC, hypertension, hyperlipidemia, diabetes duration, family history of ischemic stroke and coronary artery disease, age at diagnosis, lipid lowering medication, cardiovascular drugs, antihypertensive drugs. Analysis of variance (ANOVA) with Tukey’s test for multiple comparison was conducted to compare following values (BMI, HbA1c, FPG, LDL-C, TC, TG) and trends according to disease duration among the three age groups. Furthermore, Hosmer-Lemeshow test was performed to evaluate the goodness of fit of the logistic regression model. All analyses were conducted using SPSS version 25.0 (IBM Co., Armonk, NY, USA) All statistical tests were two-tailed and the significance level was set at *P<*0.05.

## 3 Results

### 3.1 Characteristics of study population

The baseline characteristics of the study population by strata of age at diagnosis and diabetes duration are shown in **Tables 1**, **2**, respectively. Of a total of 1765 patients, the mean (± SD) age of the cohort was 56.96 ± 10.02 years and the age at diagnosis was 52.91 ± 10.25 years, with 19.7%, 53.0% and 27.3% of patients reporting their age at diagnosis as 18−43, >44−59 and >60−74 years, respectively (**Table 1**). The mean (± SD) diabetes duration was 8.06 ± 5.66 years, with 40.0%, 29.9%, 18.5% and 11.7% of patients reporting a diabetes duration of ≤5, >5−10, >10−15 and >15 years, respectively (**Table 2**). The mean age at diagnosis of the group with the longest diabetes duration (>15 years) was significnatly lower than of the groups with shorter diabetes duration (*P<*0.001, **Table 2**). Early-onset group (aged ≤43 years) and longest diabetes duration group (>15 years) were more likely to have higher level of FPG and HbA1c, and higher family history of diabetes, compared to those diagnosed at older age group (44 to 59 and 60 to 74 years) and shorter diabetes duration (≤ 15 years) (*P<*0.001, **Tables 1**, **2**). Furthermore, compared with the younger onset age group, participants reporting diagnosed at elderly onset group were more likely to have hypertension and take antihypertensive drugs, to have coronary vascular disease and use cardiovascular drugs, to have ischemic stroke and take lipid lowering medication (P<0.001, **Table 1**). Besides, the longest diabetes duration (>15 years) were also more likely to have hypertension, ischemic stroke and coronary vascular disease compared with the shorter diabetes duration. However, participants reporting with the longer diabetes duration (10−15 years) were more likely to take antihypertensive drugs, and cardiovascular drugs (*P*<0.001, **Table 2**). Meanwhile, compared with participants reporting older age group and shorter diabetes duration, those reporting diagnosed at early-onset group and longest diabetes duration were more likely to have higher DBP level (*P<*0.001, **Tables 1**, **2**). No interaction was observed between the age at diagnosis and diabetes duration on gender, WC, lipid abnormalities, medical insurance, family history of diseases (cardiovascular disease, ischemic stroke and hyperlipidemia), glucose lowering medication and comorbidities of hyperlipidemia (all *P >*0.05) (**Tables 1**, **2**).

**Table 1:** Characteristics of the participants with diabetes mellitus stratified by age at diagnosise.

Variables (mean (SD) or N (%))	Age at diagnosis, years			
	≤43 (n=347)	44-59 (n=936)	60-74(n=482)	*P* value
Age, years	44.43(8.05)	56.27(6.62)	67.30(3.90)	<0.001
Disease duration, years	10.38(7.42)	8.29(5.37)	5.93(3.64)	<0.001
Male	181(52.2)	442(47.2)	250(51.9)	0.135
BMI, kg/m2	26.17(4.39)	25.98(3.37)	26.00(3.62)	0.709
Weight, kg	69.18(13.45)	67.69(10.84)	66.06(11.07)	<0.001
Waist circumference, cm	89.42(11.35)	89.57(9.23)	90.18(10.00)	0.454
SBP, mmHg	143(20.70)	147(20.68)	150(22.55)	<0.001
DBP, mmHg	86(12.74)	84(11.65)	80(11.60)	<0.001
FPG, mmol/L	10.36(4.00)	9.49(3.16)	8.67(2.62)	<0.001
HbA1c, %	7.97(1.99)	7.58(1.72)	7.30(1.56)	<0.001
TC, mmol/L	5.19(1.24)	5.18(1.20)	5.18(1.17)	0.995
LDL-C, mmol/L	3.07(1.07)	3.06(0.95)	3.07(0.95)	0.975
HDL-C, mmol/L	1.42(0.49)	1.42(0.40)	1.47(0.42)	0.096
TG, mmol/L	2.55(3.29)	2.34(2.60)	3.14(2.13)	0.079
Obesity	108(31.1)	222(23.8)	119(24.7)	0.025
Medical insurance	335(96.5)	911(97.3)	466(96.7)	0.681
Marital status				<0.001
Single	13(3.7)	8(0.9)	9(1.9)	
Married or lived together	323(93.1)	879(93.9)	415(86.1)	
Divorced or separated	5(1.4)	13(1.4)	8(1.7)	
Windowed	6(1.7)	36(3.8)	50(10.4)	
Education level				<0.001
Primary school or below	165(47.6)	538(57.5)	359(74.5)	
Middle school	122(35.2)	267(28.5)	83(17.2)	
Highschool or beyond	60(17.3)	131(14.0)	40(8.3)	
Total annual family income, yuan				<0.001
≤18000	51(14.7)	173(18.5)	130(27.0)	
18000 to ≤40000	138(39.8)	387(41.3)	224(46.5)	
40000 to ≤70000	76(21.9)	193(20.6)	77(16.0)	
>70000	82(23.6)	183(19.6)	51(10.6)	
Current drinking	104(30.0)	284(30.3)	102(21.2)	0.001
Current smoking	79(22.8)	196(20.9)	73(15.1)	0.01
Family history of hypertension	230(66.3)	584(62.4)	274(56.8)	0.018
Family history of diabetes	190(54.8)	443(47.3)	152(41.5)	<0.001
Family history of obesity	137(39.5)	301(32.2)	133(27.6)	0.001
Family history of cardiovascular disease	69(19.9)	178(19.0)	107(22.2)	0.365
Family history of ischemic stroke	82(23.6)	210(22.4)	107(22.2)	0.874
Family history of hyperlipidemia	94(27.1)	232(24.8)	91(18.9)	0.011
Antihypertensive drugs	103(29.7)	405(43.3)	246(51.0)	<0.001
Cardiovascular drugs	9(2.6)	67(7.2)	58(12.0)	<0.001
Lipid lowering medication	35(10.1)	151(16.1)	65(13.5)	0.019
Glucose lowering medication				0.323
Insulin	30(8.6)	121(12.9)	57(11.8)	
Oral hypoglycemic drugs	265(76.4)	690(73.7)	358(74.3)	
Comorbidities				
Coronary vascular disease	17(4.9)	60(6.4)	68(14.1)	<0.001
Hypertension	124(35.7)	469(50.1)	282(58.5)	<0.001
Ischemic stroke	23(6.6)	111(11.9)	100(20.7)	<0.001
Hyperlipidemia	213(61.4)	618(66.0)	313(64.9)	0.302

Data are Number(%) for categorical variables, and mean (SD) for continuous variables.

P values were derived from analysis of variance or Mann-Whitney U tests for continuous variables according to data distribution and χ2 test for category variables.

BMI, body mass index; SD, standard deviation; SBP, systolic blood pressure; DBP, diastolic blood pressure; FPG, fasting plasma glucose; HbA1c, glycosylated hemoglobin;TG, triglyceride; HDL-C, high-density lipoprotein cholesterol; LDL-C, low density lipoprotein cholesterol; TC, total cholesterol.

**Table 2:** Baseline characteristics by strata of diabetes duration.

Variables (mean (SD) or N (%))	Diabetes duration, years				
	≤5 (n=706)	5-10 (n=527)	11-15 (n=326)	>15 (n=206)	*P* value
**Age, years**	54.57(10.56)	56.81(9.38)	60.02(8.91)	69.67(8.98)	<0.001
**Age when diabetes first diagnosed, years**	55.38(10.53)	53.35(9.36)	51.71(8.84)	45.21(9.47)	<0.001
**Male**	369(52.3)	254(48.2)	142(43.6)	108(52.4)	0.05
**BMI, kg/m2**	26.23(3.72)	26.10(3.64)	25.82(3.30)	25.45(3.96)	0.035
**Weight, kg**	68.54(11.96)	67.61(11.66)	66.38(10.18)	66.77(11.15)	0.004
**Waist circumference, cm**	89.99(10.36)	89.95(9.55)	89.69(9.29)	88.16(9.91)	0.113
**SBP, mmHg**	146(21.50)	148(21.90)	148(20.51)	148(21.35)	0.697
**DBP, mmHg**	85(12.03)	84(12.02)	82(11.02)	80(12.70)	<0.001
**FPG, mmol/L**	8.72(2.87)	9.65(3.28)	10.05(3.45)	10.37(3.64)	<0.001
**HbA1c, %**	7.06(1.51)	7.77(1.83)	8.03(1.78)	8.17(1.75)	<0.001
**TC, mmol/L**	5.18(1.18)	5.18(1.21)	5.24(1.22)	5.06(1.21)	0.421
**LDL-C, mmol/L**	3.06(0.97)	3.04(0.96)	3.15(0.98)	3.02(0.98)	0.346
**HDL-C, mmol/L**	1.44(0.41)	1.40(0.41)	1.45(0.44)	1.48(0.44)	0.08
**TG, mmol/L**	2.36(2.67)	2.52(2.89)	2.16(2.27)	1.97(2.32)	0.048
**Obesity**	176(24.9)	140(26.6)	83(25.5)	50(24.3)	0.897
**Medical insurance**	680(96.3)	513(97.3)	321(98.5)	198(96.1)	0.231
**Current drinking**	208(29.5)	147(27.9)	83(25.5)	52(25.2)	0.469
**Current smoking**	153(21.7)	99(18.8)	56(17.2)	40(19.4)	0.343
**Family history of hypertension**	423(59.9)	326(61.9)	207(63.5)	132(64.1)	0.595
**Family history of diabetes**	268(38.0)	233(44.2)	174(53.4)	110(53.4)	<0.001
**Family history of obesity**	231(32.7)	161(30.6)	104(31.9)	75(36.4)	0.493
**Family history of cardiovascular disease**	150(21.2)	100(19.0)	64(19.6)	40(19.4)	0.231
**Family history of ischemic stroke**	160(22.7)	121(23.0)	73(22.4)	45(21.8)	0.99
**Family history of hyperlipidemia**	171(24.2)	127(24.1)	71(21.8)	48(23.3)	0.842
**Antihypertensive drugs**	261(37.0)	223(42.3)	166(50.9)	104(50.5)	<0.001
**Cardiovascular drugs**	38(5.4)	37(7.0)	39(12.0)	20(9.7)	0.002
**Lipid lowering medication**	78(11.0)	75(14.2)	59(18.1)	39(18.9)	0.004
**Glucose lowering medication**					0.386
** Insulin**	88(12.5)	60(11.4)	30(9.2)	30(14.6)	
**Oral hypoglycemic drugs**	518(73.4)	403(76.5)	244(74.8)	148(71.8)	
**Comorbidities**					
**Coronary vascular disease**	49(6.9)	38(7.2)	33(10.1)	25(12.1)	0.046
**Hypertension**	325(46.0)	253(48.0)	180(55.2)	117(56.8)	0.006
**Ischemic stroke**	69(9.8)	61(11.6)	61(18.7)	43(20.9)	<0.001
**Hyperlipidemia**	451(63.9)	339(64.3)	228(69.9)	126(61.2)	0.152

Data are Number (%) for categorical variables, and mean (SD) for continuous variables.

P values were derived from analysis of variance or Mann-Whitney U tests for continuous variables according to data distribution and χ2 test for category variables.

BMI, body mass index; SD, standard deviation; SBP, systolic blood pressure; DBP, diastolic blood pressure; FPG, fasting plasma glucose; HbA1c, glycosylated hemoglobin; TG, triglyceride; HDL-C, high-density lipoprotein cholesterol; LDL-C, low density lipoprotein cholesterol; TC, total cholesterol.

### 3.2 Different patterns of parameters and disease duration according to age at diagnosis

**Figure 2:**
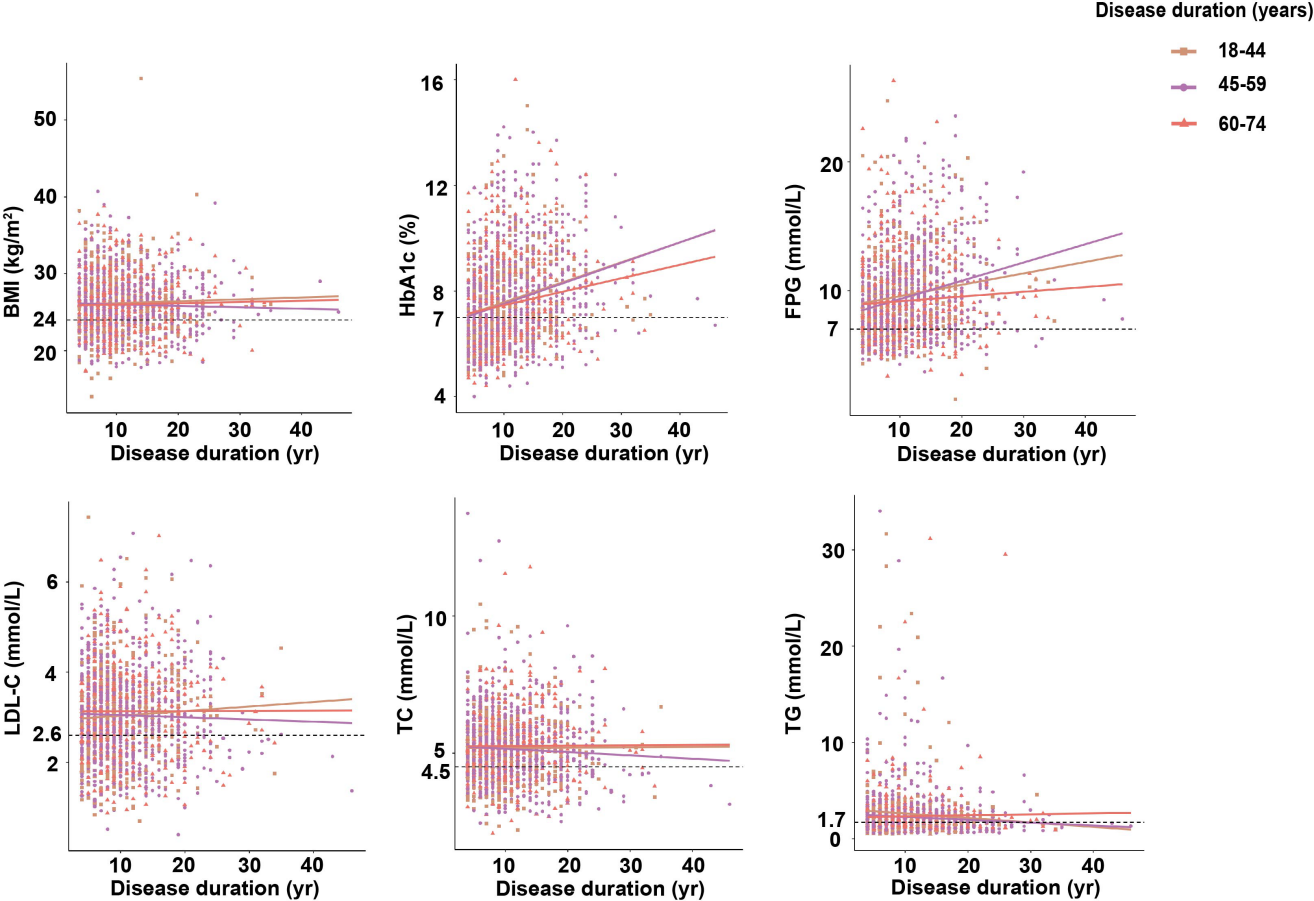
Relation between disease duration and clinical parameters according to age at diagnosis.

As shown in **Figure 2**, HbA1c and FPG increased with the duration of disease, which exceeding the maximum normal reference value (HbA1c<7.0%, FPG<7.0 mmol/L) (1). The HbA1c showed positively associated with the duration of diabetes and the degree of increase was more prominent in participants with late-onset group (R^2^ = 0.066, 44 to 59 years) than those with early-onset group (R^2^ = 0.058, *P<*0.001, ≤43 years) and elderly-onset group (R^2^ = 0.026, *P<*0.001, 60 to 74 years). The significant association was also observed in FPG, and the degree of increase was more prominent in participants reporting late-onset group (R^2^ = 0.065, *P<*0.001) than those with early-onset group (R^2^ = 0.02, *P<*0.001), while no significant association with elderly-onset group (*P* =0.17). However, the trend of increasing BMI, TC, TG, LDL-C according to disease duration was incomparable among age groups (all *P >*0.05).

### 3.3 Risk factors of diabetic macrovascular complications (coronary artery disease and ischemic stroke) in participants with DM

The prevalence of ischemic stroke was different among the three age-onset groups and four diabetes duration groups (*P*<0.001) (**Tables 1**, **2**). Coronary artery disease and ischemic stroke were significantly more common in elderly-onset group and longest diabetes duration (*P*<0.001) (**Tables 1**, **2**). With regard to risk factors associated with ischemic stroke complications in patients with DM, disease duration (OR, 1.091; 95% CI, 1.060 to 1.124; *P<*0.001), hypertension (OR, 2.729; 95% CI, 1.968 to 3.785; *P<*0.001), early-onset group (≤43 years, OR, 2.323; 95% CI, 1.367 to 3.947; *P* = 0.002), and late-onset group (44 to 59 years, OR, 5.199; 95% CI, 2.602 to 10.386, *P<* 0.001) were independently associated with the risk of ischemic stroke. Meanwhile, diabetes duration (OR, 1.080; 95% CI, 1.043 to 1.118; *P<*0.001), hypertension (OR, 2.015; 95% CI, 1.363-2.979, *P<*0.001), hyperlipidemia (OR, 1.527; 95% CI, 1.014-2.298, *P* = 0.043), family history of coronary heart disease (OR, 2.315; 95% CI, 1.592-3.365, *P<*0.001), and late-onset group (44 to 59 years old; OR, 5.001, 95% CI, 2.280-10.969, *P<*0.001) were associated with coronary artery disease. No interactions was observed between age, BMI, central obesity, and the risk of macrovascular events (all *P >*0.05) (**Table 3**).

**Table 3:** Risks factors associated with ischemic stroke and coronary disease.

Variables	Ischemic stroke		Coronary artery disease	
	OR(95%CI)	*P* value	OR(95%CI)	*P* value
Age, years	1.165(0.775-1.751)	0.463	0.903(0.555-1.468)	0.681
BMI, kg/m2	0.780(0.527-1.152)	0.212	1.066(0.660-1.720)	0.794
Central obesity	1.216(0.821-1.800)	0.329	0.974(0.604-1.570)	0.913
Hypertension	2.729(1.968-3.785)	<0.001	2.015(1.363-2.979)	<0.001
Hyperlipidemia	1.612(0.821-1.800)	0.329	1.527(1.014-2.298)	0.043
diabetes duration, years	1.091(1.060-1.124)	<0.001	1.080(1.043-1.118)	<0.001
Family history of ischemic stroke	1.286(0.923-1.793)	0.137	–	–
Family history of coronary heart disease	–	–	2.315(1.592-3.365)	<0.001
Age at DM diagnosis, years		<0.001		<0.001
≤43	2.323(1.367-3.947)	0.002	1.650(0.893-3.049)	0.11
44-59	5.199(2.602-10.386)	<0.001	5.001(2.280-10.969)	<0.001
60-74	1(reference)	–	1(reference)	–

OR, odds ratio; CI, confidence interval; BMI, body mass index; DM, diabetes mellitus.

### 3.4 Characteristics of the participants stratified by estimated ten-year ASCVD risk (%)

The baseline characteristics of the 1,423 participants stratified by estimated ten-year ASCVD were shown in **Table 4**, respectively. Overall, the mean (± SD) age of the cohort was 55.68 ± 10.01 years, the age at diagnosis was 52.03 ± 10.15 years, and the mean (± SD) diabetes duration was 7.66 ± 5.36 years, respectively (**Table 4**). According to predicted ASCVD risk, participants who were identified with high risk (≥10%) were more likely to be male (51.8% vs. 50.3% vs. 38.9%; *P* = 0.004), older (59.40 years vs. 52.91 years vs. 42.58 years; *P*<0.001), current smoking (22.4% vs. 12.1% vs. 21.1%; *P* = 0.005) and higher central obesity (82.7% vs. 67.1% vs. 59.1%; *P*<0.001), higher BMI (26.38 kg/m^2^ vs. 25.17 kg/m^2^ vs. 24.87 kg/m^2^; *P*<0.001) and WC (91.05 cm vs. 87.11cm vs. 85.25 cm; *P*<0.001), higher value of SBP (154.45 mmHg vs. 136.54 mmHg, vs. 126.90 mmHg; P<0.001) and DBP (85.42 mmHg vs. 81.30 mmHg vs. 78.68 mmHg; P<0.001), higher TC (5.28 mmol/L vs. 5.21 mmol/L vs. 4.95 mmol/L; P = 0.002) and TG level (2.63 mmol/L vs. 2.01 mmol/L vs. 1.92 mmol/L; P<0.001) compared with the lower risk groups (low risk and median risk). In addition, compared with the lower risk groups, participants who were identified with high risk were more likely to diagnose at elderly-onset group (60-74 years, 23.7% vs. 1.0% vs. 9.9%; *P*<0.001) and less likely to diagnose at early-onset group (11.5% vs. 66.7% vs. 23.4%; *P*<0.001), and more likely to have lower family history of diabetes (42.1% vs. 48.0% vs. 42.1%; *P* = 0.002) and have hypertension (59.8% vs. 5.6% vs. 22.0%; *P*<0.001) and hyperlipidemia (66.3% vs. 47.5% vs. 60.0%; P<0.001). Furthermore, compared with the lower risk groups, participants who were identified with high risk were more likely to take blood pressure medicine (50.6% vs. 20.4% vs. 5.1%; *P*<0.001), lipid lowering medication (12.9% vs. 8.9% vs. 3.5%; *P*<0.001) Besides, participants who were identified with medial risk were more likely to take cardiovascular drugs (4.6% vs. 2.0% vs. 0.5%; *P* = 0.005). Compared to those with a diabetes duration of<5 years, a diabetes duration of ≥10 years,was associated with increased estimated risk of 10-year ASCVD risk (*P* = 0.001). However, there was no evidence for FPG, HbA1c, LDL-C, current drinking, medical insurance, family history of a series of diseases (hypertension, obesity, cardiovascular disease, ischemic stroke and hyperlipidemia) and glucose lowering medication between three groups (all *P >*0.05) (**Table 4**).

**Table 4:** Characteristics of the participants with diabetes mellitus stratified by estimated 10-year ASCVD risk (%).

Variables (mean (SD) or N (%))	Estimated 10-year ASCVD risk			
	low risk (<5%)	(medial risk (5-9.9%)	high risk (≥10%)	*P* value
Age, years	42.58(7.72)	52.91(7.53)	59.40(8.37)	<0.001
Disease duration, years	6.18(4.41)	7.18(5.06)	8.13(5.57)	<0.001
Weight, kg	64.43(12.59)	66.16(11.66)	68.53(11.31)	<0.001
Waist circumference, cm	85.25(11.52)	87.11(9.63)	91.05(9.16)	<0.001
BMI, kg/m2	24.87(3.84)	25.17(3.62)	26.38(3.53)	<0.001
SBP, mmHg	126.90(13.62)	136.54(14.01)	154.45(19.85)	<0.001
DBP, mmHg	78.68(10.19)	81.30(10.15)	85.42(12.30)	<0.001
FPG, mmol/L	9.58(3.95)	9.79(3.53)	9.42(3.10)	0.231
HbA1c, %	7.57(2.01)	7.61(1.93)	7.60(1.67)	0.959
TC, mmol/L	4.95(1.04)	5.21(1.13)	5.28(1.21)	0.002
LDL-C, mmol/L	2.94(0.90)	3.14(0.97)	3.08(0.96)	0.074
HDL-C, mmol/L	1.50(0.50)	1.51(0.42)	1.41(0.42)	<0.001
TG, mmol/L	1.92(1.73)	2.01(2.06)	2.63(3.25)	<0.001
Male	77(38.9)	153(50.3)	477(51.8)	0.004
Medical insurance	191(96.5)	293(96.4)	893(97.0)	0.856
Education level				<0.001
Primary school or below	90(45.5)	169(155.6)	576(62.5)	
Middle school	59(34.8)	85(28.0)	240(26.1)	
Highschool or beyond	39(19.7)	50(16.4)	105(11.4)	
Total annual family income, yuan				<0.001
≤18000	17(8.6)	40(13.2)	214(23.2)	
18000 to ≤40000	75(37.9)	109(35.9)	410(44.5)	
40000 to ≤70000	52(26.3)	85(28.0)	156(16.9)	
>70000	54(27.3)	70(23.0)	141(15.3)	
Current drinking	47(23.7)	99(32.6)	276(30.0)	0.1
Current smoking	24(12.1)	64(21.1)	206(22.4)	0.005
Family history of hypertension	108(54.5)	179(58.9)	581(63.1)	0.057
Family history of diabetes	95(48.0)	163(53.6)	388(42.1)	0.002
Family history of obesity	70(35.4)	91(29.9)	294(31.9)	0.444
Family history of cardiovascular disease	25(12.6)	52(17.1)	184(20.0)	0.043
Family history of ischemic stroke	38(19.2)	74(24.3)	195(20.0)	0.345
Family history of hyperlipidemia	50(25.3)	71(23.4)	201(21.8)	0.546
Central obesity	117(59.1)	204(67.1)	762(82.7)	<0.001
Antihypertensive drugs	10(5.1)	62(20.4)	466(50.6)	<0.001
Cardiovascular drugs	1(0.5)	14(4.6)	18(2.0)	0.005
Lipid lowering medication	7(3.5)	27(8.9)	119(12.9)	<0.001
Glucose lowering medication				0.366
Insulin	18(9.1)	43(14.1)	112(12.2)	
Oral hypoglycemic drugs	158(79.8)	221(72.7)	682(74.0)	
Disease duration, years				<0.001
<5	107(54.0)	137(45.1)	356(38.7)	
5-10	53(26.8)	96(31.6)	287(31.2)	
10-15	27(13.6)	47(15.5)	167(18.1)	
>15	11(5.6)	24(7.9)	111(12.1)	
Age at diagnosis with DM				<0.001
20-43	132(66.7)	71(23.4)	106(11.5)	
44-59	64(32.3)	203(66.8)	510(55.4)	
60-74	2(1.0)	30(9.9)	337(23.7)	
Hypertension	11(5.6)	67(22.0)	551(59.8)	<0.001
Hyperlipidemia	11(5.6)	67(22.0)	551(59.8)	<0.001

Data are Number (%) for categorical variables, and mean (SD) for continuous variables.

P values were derived from analysis of variance or Mann-Whitney U tests for continuous variables according to data

distribution and χ2 test for category variables.

BMI, body mass index; SD, standard deviation; SBP, systolic blood pressure; DBP, diastolic blood pressure; FPG, fasting plasma glucose; HbA1c, glycosylated hemoglobin; TG, triglyceride; HDL-C, high-density lipoprotein cholesterol; LDL-C, low density lipoprotein cholesterol; ASCVD, atherosclerotic cardiovascular disease; TC, total cholesterol.

Estimated 10-year ASCVD risk.

### 3.5 Risk factors of the high risk of estimated ten-year ASCVD in participants with DM

The risk of estimated 10-year ASCVD was different among the three groups (<5%, 5%-9.9%, ≥10%: 13.9% vs. 21.4% vs. 64.7%, *P*<0.001). With regard to risk factors associated with high risk of 10-year ASCVD in participants with DM, aged over 65 (OR, 10.192; 95% CI, 6.188 to 16.788; *P*<0.001), central obesity (OR,1.992; 95% CI, 1.361 to 2.914; *P*<0.001), hypertension (OR, 18.816; 95% CI, 7.286 to 48.592; *P<*0.001), hyperlipidemia (OR, 1.366; 95% CI, 1.007 to 1.852; *P* = 0.045) were independently associated with the high risk of estimated ten-year ASCVD in participants with DM. Compared to those with a diabetes duration of ≤15 years, a diabetes duration of >15 years was associated with increased high risk of estimated ten-year ASCVD in in participants with DM (OR, 1.976; 95% CI, 1.156 to 3.377; *P* = 0.013). Compared to those without using cardiovascular and antihypertensive drugs, treatment with using above drugs were associated with increased high risk of estimated ten-year ASCVD (OR, 5.184, 95% CI, 1.976 to 13.601; *P* = 0.001; OR 2.780; 95%CI, 1.047 to 7.384; *P* = 0.040). No interactions was observed between BMI, family history of ASCVD, age at diagnosis, and patients with diabetes duration 5–10 years, 10–15 years and the age at diagnosis (all *P >*0.05) (**Table 5**).

**Table 5:** Risk factors of the high risk of estimated 10-year ASCVD in participants with DM.

Variables	High risk of estimated 10-year ASCVD		
	OR	95%CI	P value
Age, years	10.192	6.188-16.788	<0.001
BMI, kg/m2	1.29	0.926-1.796	0.132
Central obeity	1.992	1.361-2.914	<0.001
Hypertension	18.816	7.286-48.592	<0.001
Hyperlipidemia	1.366	1.007-1.852	0.045
Diabetes duration, years			0.061
<5	1	reference	–
5-10	1.368	0.978-1.912	0.067
10-15	1.361	0.895-2.071	0.150
>15	1.976	1.156-3.377	0.013
Lipid lowering medication	1.028	0.606-1.746	0.917
Cardiovascular drugs	5.184	1.976-13.601	0.001
Antihypertensive drugs	2.780	1.047-7.384	0.04
Family history of ASCVD	1.025	0.752-1.396	0.876
Age at diagnosis, years			0.985
20-43	1	reference	–
44-59	1.053	0.729-1.520	0.784
60-74	1.046	0.681-1.606	0.838

ASCVD, atherosclerotic cardiovascular disease; OR, odds ratio; CI, confidence interval; BMI, body mass index; DM, diabetes mellitus.

P < 0.05.

## 4 Discussions

In this population-based study, we have described the risks for diabetes related complications, and how this varies by attained age, BMI, WC, hypertension, hyperlipidemia, duration of diabetes, age at diagnosis, and family history of ischemic stroke and coronary heart disease. Several factors increase the risk of heart attack, such as hyperglycaemia, obesity, abnormal cholesterol levels, hypertension, and smoking (20).

Participants who were diagnosed with early-onset (age ≤43 years) had higher FPG and HbA1c level than those diagnosed at older ages (aged 44 to 74 years). Most complications in patients with DM are associated with hyperglycaemia, which are important components of metabolic syndrome and may be the early manifestations of insulin resistance (25). Hyperglycaemia can induce oxidative stress in the vasculature, leading to disruption of the normal endothelial function and impaired relaxation of the arterial vascular smooth muscle cells (26). Hyperglycaemia also leads to excessive production of advanced glycation end products and cytokines, which cause activation of adhesion molecules and thickening of intima media (27). Vascular endothelial dysfunction and adhesion activation are also related to hypertension (28), dyslipidemia and diabetic cardiomyopathy (29). Individuals with early-onset diabetes have accompanying metabolic risk factors similar to, or even worse than, those of individuals with late-onset diabetes. For early-onset patients with diabetes, effective glycemic control may contribute to hyperglycaemic episodes, treatment of dyslipidemia, hypertension, obesity, therefore, counteract both microvascular and macrovascular complications of diabetes. Although glucose levels can be controlled through diet, exercise and medications including insulin and oral hypoglycemic agents, many patients continue to undergo numerous life-threatening complications following glucose normalization, suggesting the persistent detrimental effects of high glucose exposure through metabolic memory even after glycaemic control has been established (30, 31). Moreover, the landmark Diabetes Control and Complications Trial (DCCT) confirmed that the damage of hyperglycaemia to microvessels can be delayed for a long time after intensive glycaemic control (32).

Several studies have shown that diabetes duration leads to atherosclerotic lesions, including intimal thickness and thin cap fibroatheromas, which contribute to the deleterious effects on small and large vessels and lead to the development of cardiovascular disease and mortality (12, 33, 34). Further study suggested that diabetes duration during adulthood was associated with coronary artery calcified plaque and left ventricular systolic and diastolic dysfunction in later life, suggesting that the cumulative exposure to chronic hyperglycemia may lead to increased risk of atherosclerosis and impaired cardiac function (35). Our study identified that increasing duration increased the high risk of estimated ten-year ASCVD. These studies are backed up by data from a large population based study from the UK Biobank, which identified that duration of diabetes was independently associated with a greater risk of CVD, myocardial infarction and stroke (36). Further study takes the findings to a broader population based scale where they showed that increasing duration and late-stage complications of DM increase the associated risk of infective endocarditis (37).

We also observed hypertension and hyperdemia were independent risk factors for high risk of estimated 10-year ASCVD in patients with diabetes. As highlighted in the study, ASCVD was prone to occur in the patients with the agedness, central obesity, longer diabetes duration, hypertension and hyperlipidemia. Previous study found that diabetic dyslipidemia precedes T2DM by several years, and lipid abnormalities were associated with an increased risk of CVD (38). Some scholars emphasized that diabetes patients are commonly accompanied by lipid metabolism disorder and hyperlipidemia due to the dysfunction of insulin biological regulation (39). On the one hand, insulin resistance can lead to blood glucose fluctuation and chronic hyperglycemia, which in turn triggers oxidative stress and increases the expression of pro-inflammatory factors and pro-coagulant factors that leads to cell damage (40). On the other hand, insulin resistance can induce an imbalance in lipoproteins profle alterations that contributes to the development of dyslipidemia and the lipid triad (41). The abnormal association between insulin resistance and endothelial signal conduction disorder leads to inflammation, which further disrupts the balance between endothelial vasodilator and vasoconstrictor mechanisms, and leads to the formation of atherosclerotic plaque (40, 42). New therapies focused on decreasing insulin resistance may be investigated as a potential future therapeutic target for mitigating both CVD and atherosclerotic plaque generation.

Compared to those patients with DM who have’t been diagnosed as ASCVD, the high risk of estimated 10-year ASCVD in DM patients treatment with antihypertensive drugs is estimated to increase up to 3-times, and the high risk level is 5-times higher than without using cardiovascular drugs. It’s well known that both antihypertensive drugs and cardiovascular drugs are perfect preventive medications for ASCVD in high-risk patients with DM. Obviously, we may arrive at a conclusion that high-risk population are more willing to take antihypertensive drugs and cardiovascular drugs under medical advice. These findings advance the arguments for wider and earlier use of China-PAR model in this population and provide important practical value for the prevention of cardiovascular diseases in diabetic patients. However, the data of patients using cardiovascular drugs in the analysis model is so small that can’t reflect the real world impact of all diabetes patients after receiving cardiovascular drug treatment.

The patients with early-onset DM were characterized by a higher level of BMI and weight, and were more likely to have obesity than older-onset DM. As an important indicator to reflect the obesity, BMI can evaluate the severity of insulin resistance in obese DM patients. Obesity increased the risk for development of DM and may lead to an earlier age of diagnosis (43, 44). Our analysis is consistent with these findings. The significant proportion of metabolic disorders and insulin resistance among patients with early-onset DM, and the risk for development of macrovascular complications from diabetes is increased among obese individuals with DM (43). Therefore, it is suggested that early-onset DM patients should repair metabolic disorders and ameliorate insulin resistance by reducing weight and BMI as soon as possible.

The results of the present study show that early-onset age of DM increases the risk of macrovascular complications later in life. Compared with late-onset DM, people with early-onset DM have a significantly higher risk of developing macrovascular complications, especially ischemic stroke. Our findings are consistent with those of two previous studies (4, 45). It was found in previous studies that patients diagnosed with early-onset (20-39 years) T2DM had a higher risk of developing cardiovascular disease and a higher cardiac 10-year expected risk than patients with late-onset T2DM (46). These studies all suggested that the impending increase in average duration of diabetes in people with T2DM is likely to increase the burden of coronary artery disease and ischemic stroke. The groups with younger age at diagnosis (age ≤59 years) was independently associated with the risk of macrovascular complications compared to the elderly-onset group (60 to 74 years). These results were similar to other studies. Observational studies showed that patients with younger age at diabetes diagnosis was associated with higher risk of vascular disease (47). And cardiovascular complications were more common in patients with early-onset T2DM at any given age (48). Therefore, early and sustained interventions are essential to delay T2DM onset, and improve blood glucose levels, and cardiovascular risk profiles of those already diagnosed. However, other studies argued that patients with early-onset diabetes are more likely to have microvascular complications than macrovascular complications (11). In the study, the increase in comorbidities of coronary vascular disease and ischemic stroke with age at diagnosis was more pronounced in the elderly-onset (60 to 74 years) than those with younger onset (age ≤59 years). Both age at diagnosis and duration of diabetes affected the risk for coronary artery disease and ischemic stroke, but the relative impact was different for each. For ischemic stroke, the incidence risk was primarily driven by disease duration and age at diagnosis, which led to the relative risk of people with different diabetes duration at a given age. However, these relative risks were smaller for coronary artery disease, indicating less of an effect of duration of diabetes and more so of age at diagnosis than for ischemic stroke.

Our findings should be interpreted in light of the strengths and limitations of our study. The strengths of this work include that data were derived from a large, multi-center and multi-level design cohort of patients with diabetes, and had comprehensive clinical implications for diabetes complications. our study found that people with early-onset diabetes had a high risk of complications, indicating diabetes-related complications will increasingly occur in people of working age, which may result in substantial healthcare burden. It is essential to intensified efforts to prevent these complications. We first used the China-PAR project to predict the ten-year ASCVD in the study, which will help to improve primary prevention of macrovascular complications. The China-PAR project developed based on the recent epidemics of CVD and risk factors will be better to identify high risk individuals with appropriately predicted risk probability, and to evaluate ASCVD outcome with standardized review process, using the same diagnosis criteria across cohorts.

The present study had some limitations. First, information on coronary heart disease and cerebrovascular disease was collected from the questionnaire, which is similar to most other epidemiological studies (49). However, it is not practical to measure coronary heart disease and cerebrovascular disease by equipment in a large-scale study. Second, previous studies show that younger age at diabetes diagnosis was associated with high risk of macrovascular and microvascular disease (47). The diabetes duration has been thought to be an noteworthy factor, absent of the ability to quantify the effects of duration of diabetes after the diagnosis of diabetes. Longer periods of follow-up may be need to be carried out among participants. It is a great pity that numbers of clinical events and kinds of complications were present in small amounts. Patients were grouped according to their age at onset, but for asymptomatic patients, the age onset and disease duration might be underestimated. However, in view of the similarity between our cohort and the cohort of patients with diabetes observed by other teams, we still believe that our findings are somewhat universal and comparable among onset-age groups (10, 12, 47). Furthermore, this was a cross-sectional study in four cities that does not represent broad sections of the people with diabetes at different region cross the country. In addition, several limitations should be addressed in our study when using the China-PAR modeling. Firstly, the project is aimed to develop and validate 10-year risk prediction equations for ASCVD from all Chinese participants, not diabetes patients. Thus, cautions should be used and training among physicians is required to communicate predicted 10-year risk of ASCVD to patients when the modeling are applied in prevention practice. Secondly, further investigation is warranted to examine whether the 10-year risk prediction modeling could have good performance in large-scale cohorts with short durations of follow-up.

## 5 Conclusions

The present study demonstrated that higher FPG and HbA1c according to disease duration were observed in patients diagnosed at early-onset age than those diagnosed at older age. Age at diagnosis and diabetes duration, hypertension and hyperlipidemia were associated with an increased risk of macrovascular events and high risk of ten-year ASCVD prediction. Further prospective cohort studies are necessary to examine our findings in large-scale populations.

## Data availability statement

The original contributions presented in the study are included in the article/supplementary material, further inquiries can be directed to the corresponding author/s.

## Ethics statement

The studies involving human participants were reviewed and approved by the Ethical Review Committee (Approval No: 2018-010). The patients/participants provided their written informed consent to participate in this study. Written informed consent was obtained from the individual(s) for the publication of any potentially identifiable images or data included in this article.

## Author contributions

QZ, HH, and FD designed and proposed the study. XY, JZ, XZ, TJ, and YZ collected the patients’ data. XY prepared and drafted the manuscript. JZ and XZ analyzed and illustrated the data. YZ and TJ revised the paper. All authors have read and approved the manuscript. In addition, we confirm that all listed authors meet the authorship criteria and that all authors are in agreement with the content of the manuscript. All authors contributed to the article and approved the submitted version.
